# Water Extract of Polygonati Rhizoma Ameliorates Obesity-Related Skeletal Muscle Atrophy in Mice and C2C12 Myotubes

**DOI:** 10.3390/nu18030429

**Published:** 2026-01-28

**Authors:** Haifeng Shao, Yang Wang, Yong-Ki Park, Hyo Won Jung

**Affiliations:** Department of Herbology, College of Korean Medicine, Dongguk University, Gyeongju 38066, Republic of Korea; shf326904@163.com (H.S.); wy1997ere@163.com (Y.W.);

**Keywords:** obesity-related muscle atrophy, sarcopenic obesity, Polygonati Rhizoma, inflammation, ubiquitin-proteasome system, myogenic differentiation

## Abstract

**Background:** Sarcopenic obesity (SO) is a metabolic myopathy characterized by the coexistence of obesity and decline of muscle mass and function. Obesity-related muscle atrophy represents a central pathological feature of this condition. Polygonati Rhizoma is widely used as a dietary herb with tonic effects in traditional Asian medicine. This study aims to investigate the effects and underlying molecular mechanisms of the water extract of Polygonati Rhizoma (WPR) on obesity-related muscle atrophy. **Methods:** The effects and potential mechanisms of WPR were explored using an obesity-induced muscle atrophy (OIMA) mouse model, palmitic acid (PA)- or lipopolysaccharide (LPS)-induced myotube atrophy models, and a myogenic differentiation model in C2C12 cells. **Results:** In OIMA mice, WPR attenuated obesity-related skeletal muscle atrophy and improved muscle strength and endurance. In the gastrocnemius muscle, WPR-treated mice showed lower levels of oxidative stress and inflammation, increased markers of mitochondrial biogenesis, and an improved balance between protein synthesis and degradation. In PA- or LPS-induced myotube atrophy models, WPR treatment suppressed the ubiquitin–proteasome system (UPS)-mediated proteolysis and NFκB/MAPK-related inflammatory signaling. In addition, WPR promoted myogenic differentiation in C2C12 myoblasts, which was associated with regulation of the p38 MAPK/MyoD/Myogenin axis. **Conclusions:** Our study suggests that WPR exerts a potential mitigating effect on obesity-related muscle atrophy, and this effect may be associated with the modulation of skeletal muscle inflammatory signaling, mitochondrial function, and protein metabolic balance. These findings are exploratory and provide mechanistic clues for future research aimed at developing potential intervention strategies for obesity-related muscle atrophy.

## 1. Introduction

Sarcopenic obesity (SO) is a metabolic myopathy, which is characterized by reduced skeletal muscle mass and strength with excessive lipid accumulation [1]. It mainly affects elderly people who are vulnerable to various metabolic diseases and often causes adverse outcomes such as impaired mobility, frailty, and high risk of falls and even death [2,3,4]. With rapid economic development and growing social stress, high-sugar and high-fat diets that provide immediate pleasure have become more prevalent, making SO an increasingly major global public health concern [5]. The prevalence of SO was reported to be about 11% among individuals aged over 60 years, increasing to 16.7% among those aged over 80 years [6,7].

Obesity-related muscle atrophy and functional impairment are recognized as one of the core pathological components of SO. Long-term high-fat diets and lack of exercise may lead to insulin resistance and ectopic lipid deposition in skeletal muscle, even if diabetes has not yet been diagnosed. This creates a lipotoxic microenvironment within skeletal muscle, accompanied by the over-accumulation of mitochondrial ROS (mtROS) and activation of inflammatory signaling, which inhibits protein synthesis and enhances protein degradation [8,9,10,11]. These changes promote each other, creating a vicious cycle of lipotoxicity, oxinflammation, and metabolic imbalance, resulting in reduced muscle mass and decreased muscle strength.

Recently, interest in muscle health and concerns about sarcopenia have increased, and pharmacological interventions have also gained attention. In SO, various treatments such as vitamin D and calcium supplementation, testosterone therapy, and selective androgen receptor modulators are being tried, but their clinical efficacy remains controversial, and concerns about long-term safety and consistent therapeutic outcomes persist [4,12]. However, developing safe and multifunctional products from dietary or natural sources has become an important direction for the prevention and treatment of SO.

Polygonati Rhizoma (Huangjing), the dried rhizome of *Polygonatum sibiricum* Redouté, is a traditional edible and medicinal plant widely used in East Asia. It serves both as a health-promoting food and a classical tonic in traditional Korean and Chinese medicine [13]. Recent pharmacological research has shown that this herb contains abundant polysaccharides, saponins, and flavonoids, which collectively contribute to its antidiabetic, antioxidant, anti-inflammatory, and mitochondrial-protective activities [14,15]. Recent evidence indicates that both the aqueous extract and the *Polygonatum sibiricum* polysaccharide (PSP) of PR are reported to exert anti-obesity effects and suppress the development of obesity-related liver disorders [16,17], and alleviate muscle atrophy associated with aging or cancer cachexia by improving mitochondrial function and regulating gut microbiota [18,19,20,21,22,23]. However, the reports on the anti-SO effects of PR are still limited, and the roles of the WPR in modulating obesity-related skeletal muscle inflammation, mitochondrial dysfunction, and protein turnover imbalance have not been reported. Furthermore, the potential regulatory role of WPR in myogenic differentiation remains unclear. Given its historical safety profile as a medicinal–edible herb, PR represents a promising candidate for long-term nutritional interventions against obesity-related muscle atrophy, offering a distinct advantage over purely pharmacological herbs [13].

Accordingly, this study was an exploratory and hypothesis-generating investigation to explore the effects of WPR on obesity-related muscle atrophy and to provide mechanistic clues for future causal validation studies. In this study, the effects of WPR on obesity-related muscle loss and functional impairment were evaluated based on an obesity-induced muscle atrophy (OIMA) mouse model by behavior test, muscle mass, and CSA. Moreover, the underlying molecular mechanisms of the anti-atrophy effects of WPR under lipotoxic and inflammatory conditions were investigated in palmitic acid (PA)- or lipopolysaccharide (LPS)-stimulated C2C12 myotube atrophy models, respectively. In addition, the regulatory effect of WPR on the myogenic differentiation was also evaluated during horse serum-induced differentiation of C2C12 myoblasts.

## 2. Materials and Methods

### 2.1. Preparation of WPR

The raw materials of PR were purchased from Dongui Herb Co., Ltd. (Pocheon, Republic of Korea) and authenticated by Professor HW Jung at Dongguk University College of Korean Medicine (DUCKM). For extraction, the dried rhizomes (200 g) were refluxed twice with distilled water (2000 mL) for 2 h each time. After filtration, the extract solution was concentrated at 60 °C under reduced pressure and lyophilized in a freeze-dryer (IlShin Lab Co., Ltd., Jeonju, Republic of Korea) at −80 °C to produce a dry powder. Finally, 134.64 g of WPR powder was obtained (yield: 67.32%). The PR and WPR samples were kept at −20 °C in the College of Korean Medicine, Dongguk University.

### 2.2. Preparation of Animal Model

Male C57BL/6 mice (8 weeks old, weighing about 25 g) were obtained from Hana Biotech Inc. (Pyeongtaek, Republic of Korea) and were housed in controlled environmental conditions at 23 ± 2 °C with a 12 h light/dark cycle with free access to chow and water. Animal management, drug administration, and monitoring of basic physiological indicators were all carried out by experimenters who were unaware of the actual grouping of the animals. All procedures were approved and conducted under the guidelines issued by the Institutional Animal Care and Use Committee (IACUC) of Dongguk University (approval No. IACUC-2024-07).

**Figure 1:**
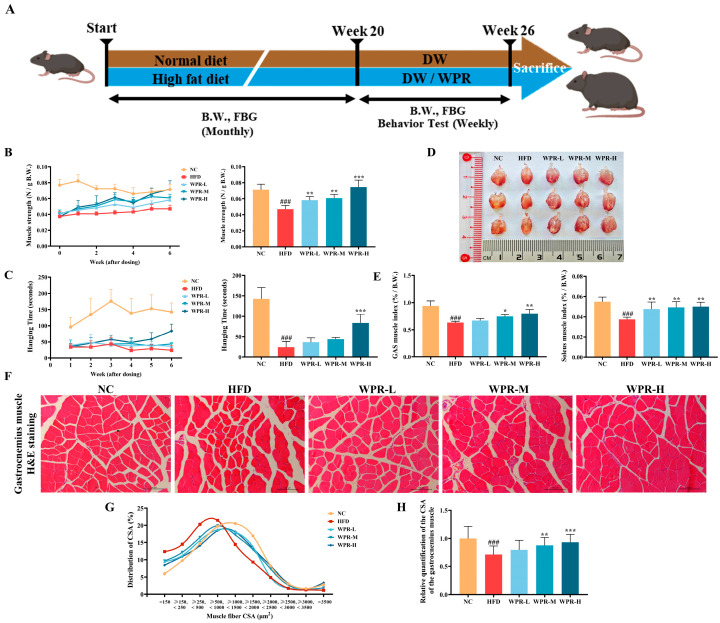
WPR administration attenuates muscle mass loss and improves muscle function in mice with OIMA. (**A**) Preparation of OIMA mouse model and experimental design. Behavior test of grip strength (**B**) and hanging time (**C**) for 6 weeks. (**D**) Representative images of GAS. (**E**) Comparisons of mass indices of GAS and SOL among groups (normalized to B.W.). (**F**) Representative images of GAS sections with H&E staining. Comparisons of the distribution curves (**G**) and the mean values (**H**) of CSA. All data are presented as the mean ± SD (*n* = 6). The *p* values were defined as follows: ^###^ *p* < 0.001 vs. the NC group; * *p* < 0.05, ** *p* < 0.01, and *** *p* < 0.001 vs. the HFD group. NC: normal control; B.W.: body weight; HFD: high-fat diet; WPR: water extract of Polygonati Rhizoma; SOL: soleus; CSA: cross-sectional area.

After acclimation, mice were randomly assigned to five groups (*n* = 6): the normal control (NC) group received a standard chow diet (#2018S, Envigo, Indianapolis, IN, USA); and the high-fat diet (HFD) group, WPR-low-dose (WPR-L) group, WPR-middle-dose (WPR-M) group, and WPR-high-dose (WPR-H) group received a high-fat diet (#D12492, Research Diets, New Brunswick, NJ, USA) for 20 weeks without drug treatment (Figure 1A).

Body weight (B.W.) and fasting blood glucose (FBG) were monitored every 4 weeks. From week 21, mice received daily oral administration of the treatments for 6 weeks (the NC and HFD groups were given distilled water; the WPR-L, WPR-M, and WPR-H groups received 0.5, 1, and 2 g/kg WPR administration, respectively). After overnight fasting, mice were anesthetized in a euthanasia chamber with 5% isoflurane (mixed gas: 25% O_2_ and 75% N_2_O). The serum samples, gastrocnemius (GAS), soleus (SOL), inguinal and epididymal white adipose tissues (iWAT and eWAT), as well as interscapular brown adipose tissue (BAT), were collected and promptly kept at −80 °C for subsequent analyses.

### 2.3. Grip Strength Test and Hanging Test

The mouse grip strength was tested using a digital grip strength meter (#FGJN-5, Jeongdo Bio Co., Ltd., Seoul, Republic of Korea) from week 21. Mice were allowed to hold a metal grid, after which they were gently pulled backward by the tail in a horizontal direction until they released their grip. Three measurements were taken per mouse, and the average value was used. Grip strength was normalized to body weight for group comparison.

The hanging test was conducted from week 21. Mice were allowed to grasp a metal grid securely with all four limbs, after which the grid was inverted to start the test. The latency of fall was recorded as a measure of muscle endurance. Each mouse performed three trials, and the mean value was used for analysis.

All behavioral tests and analyses were conducted by experimenters who were unaware of the actual group assignments.

### 2.4. Histopathological Analysis

The GAS, liver, and kidney tissues were fixed in 4% paraformaldehyde (PFA; #CBPF-9004, CHEM-BIO, Seoul, Republic of Korea) at 4 °C overnight. Tissues were then dehydrated through a graded ethanol series, cleared with xylene, embedded in paraffin, and then sectioned at 4 μm using a Leica microtome (Wetzlar, Germany). After deparaffinization and rehydration, tissue sections were subjected to hematoxylin and eosin staining. Images were acquired by experimenters unaware of the actual experimental groupings using a light microscope (Leica, Wetzlar, Germany), and the muscle fiber cross-sectional area (CSA) was quantified using ImageJ software (version 1.52a, https://imagej.net/ij/, accessed on 1 May 2025).

### 2.5. MDA and SOD Activity Assay

MDA and SOD levels in GAS were analyzed by an experimenter unaware of the actual experimental groupings using TBARS (#DG-TBA200) and SOD (#DG-SOD400) kits (Dogenbio, Seoul, Republic of Korea). GAS tissue (50 mg) was homogenized on ice with PBS + 1% BHT or sucrose buffer for MDA and SOD assays, respectively. After centrifuging at 10,000 rpm for 15 min at 4 °C. The obtained supernatant was processed in accordance with the manufacturers’ protocols.

### 2.6. Enzyme-Linked Immunosorbent Assay (ELISA)

IL-1β levels in the GAS tissues were measured by an experimenter unaware of the actual experimental groupings using a mouse IL-1β ELISA kit (#EM0109, Finetest, Wuhan, China) after normalizing the protein lysates to an equal protein level.

### 2.7. Quantitative Real-Time PCR Analysis

Total RNA was extracted from GAS tissues and C2C12 cells using the RNeasy Mini Kit (#74104, QIAGEN, Germantown, MD, USA). The RNA was reverse-transcribed into cDNA with the ReverTra Ace qPCR RT Master Mix (#FSQ-201, TOYOBO, Osaka, Japan). Quantitative real-time PCR was performed on a CFX96 Detection System (Bio-Rad, Hercules, CA, USA) using the TOPreal SYBR Green qPCR PreMIX (#RT500M, Enzynomics, Daejeon, Republic of Korea). Gene expression levels were analyzed by the 2^−ΔΔCT^ method, and *gapdh* served as the internal control. Primer sequences are provided in Supplementary Table S1.

### 2.8. Western Blotting Analysis

Protein lysates were prepared with RIPA buffer (#78510, Thermo Scientific, Waltham, MA, USA), and equal quantities were analyzed by SDS-PAGE and Western blotting. After primary and secondary antibody incubation, signals were detected by ECL (#1705061, Bio-Rad, USA) in a Gel Imaging System (Bio-Rad, USA) and quantified using ImageJ based on at least three independent biological replicates. β-Actin or GAPDH was used as the internal control, and antibody details are provided in Supplementary Table S2.

### 2.9. Cell Culture and Treatment

C2C12 myoblasts (#CRL-1772, ATCC, Manassas, VA, USA) were cultured in Dulbecco’s Modified Eagle Medium (DMEM, #11965-092, Gibco, Grand Island, NY, USA) supplemented with 10% fetal bovine serum (#16000-044, Gibco) and 1% penicillin–streptomycin (#15140-122, Gibco) in a humidified incubator at 37 °C with 5% CO_2_. Differentiation was induced at 90% confluence by switching the culture medium to differentiation medium containing 2% horse serum (HS, #16050-130, Gibco). The differentiation medium was refreshed every 36 h; day 0 was defined as the time point of confluence prior to medium replacement, and myotubes formed by day 5.

To evaluate whether WPR alleviates PA- or LPS-induced myotube atrophy, myotubes (day 5) were pretreated with WPR for 2 h before being exposed to PA (0.5 mM; #SLCF9094, Sigma-Aldrich, St. Louis, MO, USA; vehicle: 1% fatty acid-free BSA, #013-15143, Wako, Osaka, Japan) or LPS (1 μg/mL, #L4516, Sigma-Aldrich) for 24 h.

To evaluate the promoting effect of WPR on myogenic differentiation, C2C12 cells were treated with WPR beginning at day 0 (after medium change). Cells were harvested at designated time points during differentiation (days 1, 3, and 5) for further analyses.

### 2.10. Cell Viability Assay

To evaluate PA-induced cytotoxicity, C2C12 myotubes (day 5) were treated with PA (0.125, 0.25, 0.5, and 0.75 mM) for 24 h in 96-well plates, followed by measurement of cell viability. To assess the cytotoxicity of WPR, C2C12 cells were seeded in 96-well plates (100 μL per well, 1 × 10^5^ cells/mL), changed with differentiation medium after reaching 90% confluence, and treated with WPR (0.1, 0.2, 0.4, 0.8, and 1.6 mg/mL). The cell viability was determined on day 5. All viability measurements were performed using the EZ-Cytox cell viability kit (#EZ500, Dogenbio, Seoul, Republic of Korea) following the manufacturer’s protocol.

### 2.11. Immunocytochemistry

For immunocytochemistry, C2C12 cells were cultured and treated in 12-well plates as described. After fixing with 4% PFA for 15 min, cells were permeabilized with 0.5% Triton X-100 for 5 min at room temperature. Cells were then blocked with 0.5% BSA for 2 h, followed by incubation with the primary antibodies overnight at 4 °C. After washing, cells were incubated with secondary antibodies and counterstained with DAPI (#1816957, Thermo Fisher Scientific, Waltham, MA, USA) at room temperature in the dark. Fluorescent signals were captured by an experimenter unaware of the actual experimental groupings using a DM2500 fluorescence microscope (Leica, Germany) and quantified using ImageJ software.

### 2.12. Oil Red O Staining

Intracellular lipid accumulation in the PA-stimulated C2C12 myotubes was evaluated using an Oil Red O staining kit (#G1262, Solarbio, Beijing, China) after treatment. After fixing with 4% PFA, cells were stained with Oil Red O, destained with 60% isopropanol, and counterstained with Mayer’s hematoxylin. Images were acquired by an experimenter unaware of the actual experimental groupings using a light microscope, and lipid accumulation was quantified using ImageJ software.

### 2.13. Ultra-High-Performance Liquid Chromatography–Quadrupole Time-of-Flight Mass Spectrometry (UHPLC-Q-TOF-MS/MS)

WPR (40.36 mg) was dissolved in 1.0 mL of 50% methanol, ultrasonicated for 10 min, centrifuged, and the supernatant was subjected to UHPLC–Q-TOF–MS/MS analysis. Separation was carried out on a Waters ACQUITY UPLC HSS T3 column using acetonitrile and 0.1% formic acid in water at 0.3 mL/min, 35 °C, and a 5 µL injection volume under the described gradient conditions. Mass spectrometric data were acquired on an Agilent 6546 Q-TOF (ESI+, ESI−; *m*/*z* 100–1700; stepped CE 10/20/40 eV; Agilent Technologies, Santa Clara, CA, USA), and compound identification was performed using MassHunter Workstation (version B.08.00, Agilent Technologies, Santa Clara, CA, USA) with PCDL and the Polygonatum phytochemical databases, followed by fragment validation with SIRIUS. Detailed information can be found in the Supplementary Data.

### 2.14. Statistical Analysis

All data were analyzed using GraphPad Prism 8.0 (GraphPad Software, San Diego, CA, USA). Results are presented as the mean ± standard deviation (SD). Differences among groups were assessed by one-way analysis of variance (ANOVA) followed by Tukey’s multiple comparisons test. A *p* value < 0.05 was considered statistically significant. Given the exploratory nature of the study and the limited sample size, *p*-values are reported as descriptive indicators of group differences rather than as definitive evidence of statistically powered hypothesis testing.

## 3. Results

### 3.1. Effects of WPR on the Muscle Mass and Function in Mice with OIMA

To evaluate the effects of WPR on the muscle dysfunction caused by obesity-related muscle atrophy, mice first received a high-fat diet for 20 weeks to induce obesity, followed by a 6-week treatment (Figure 1A). As shown in Figure 1B, grip strength was consistently lower in the HFD-fed mice compared with the mice in the NC group throughout the 6-week period, whereas WPR treatment gradually restored muscle strength. Notably, the WPR-H group showed an improvement compared with the HFD group at week 6. Likewise, in the hanging test, the HFD group exhibited reduced latency to fall compared with the NC group throughout the 6-week period (Figure 1C). However, WPR administration prolonged the hanging time, particularly in the WPR-H group at week 6.

In the mass of muscle tissues (Figure 1D,E), the HFD group showed lower muscle mass indices of both GAS and SOL. However, they were significantly increased in the WPR-treated groups (*p* < 0.01 in the WPR-H group).

H&E staining of GAS sections demonstrated clear histopathological evidence of muscle atrophy in the HFD group, including reduced myofiber diameter and disrupted fiber organization (Figure 1F). While WPR administration improved this damage with organized fiber alignment and larger CSA. Quantitative analysis revealed that the HFD group exhibited a shift toward smaller fibers of the CSA distribution (Figure 1G). However, WPR administration shifted the distribution toward larger fibers and increased the mean CSA (Figure 1H). Collectively, these findings indicate that WPR attenuated muscle loss and functional impairment in the OIMA mouse model. Subsequent analyses were performed to explore potential mechanisms underlying these phenotypic changes.

### 3.2. Effects of WPR on the Oxidative Stress and Inflammation in the GAS of Mice with OIMA

**Figure 2:**
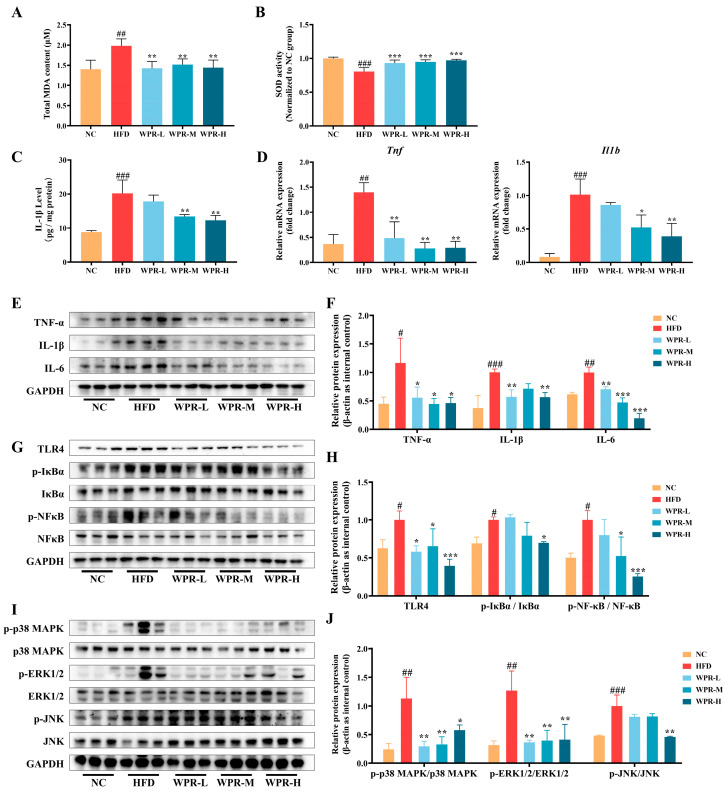
WPR administration alleviates oxidative stress and inflammation in the GAS of mice with OIMA. (**A**–**C**) Comparison of MDA levels (**A**), SOD activities (**B**), and IL-1β levels (**C**) in the GAS among groups (*n* = 6). (**D**) Relative mRNA expression levels of *Tnf* and *Il1b* in the GAS among groups. (**E**–**J**) Representative Western blot images (**E**,**G**,**I**) and quantitative analyses (**F**,**H**,**J**) of TNF-α, IL-1β, IL-6, TLR4, phospho- and total-IκBα, NF-κB p65, p38 MAPK, JNK, and ERK1/2 in the GAS of each group (*n* = 3). All data are presented as the mean ± SD. The *p* values were defined as follows: ^#^ *p* < 0.05, ^##^ *p* < 0.01, and ^###^ *p* < 0.001 vs. NC group; * *p* < 0.05, ** *p* < 0.01, and *** *p* < 0.001 vs. HFD group. NC: normal control; HFD: high-fat diet; WPR: water extract of Polygonati Rhizoma.

To evaluate the impact of WPR on oxidative stress and inflammatory responses in the GAS under obesity conditions, MDA levels, SOD activities, and the expression of inflammatory cytokines and associated signaling molecules were measured. As a result, significantly increased oxidative stress was shown in the HFD group, as evidenced by elevated MDA levels (Figure 2A) and reduced SOD activity (Figure 2B) compared with the NC group. However, WPR-treated mice exhibited lower levels of MDA and higher levels of SOD activity. In addition, IL-1β levels in GAS tissues were markedly elevated in the HFD group. However, WPR-treated groups exhibited lower levels of IL-1β (Figure 2C).

Western blot analysis (Figure 2E,F) showed increased protein expression of TNF-α, IL-1β, and IL-6 protein levels in the GAS, but the WPR-H group displayed lower expression of TNF-α, IL-1β, and IL-6. Consistently, qRT-PCR analysis showed that the mRNA levels of *Tnf* and *Il1β* were significantly upregulated in the HFD group, whereas WPR administration reduced their expression (Figure 2D).

To explore the changes in inflammatory signaling pathways, we detected the activation of TLR4/NF-κB and MAPK pathways in GAS (Figure 2G–J). As a result, HFD feeding led to a significant upregulation of TLR4 expression together with enhanced phosphorylation of IκBα and NF-κB p65. However, the WPR-H group showed lower TLR4 expression and reduced phosphorylation of IκBα and NF-κB p65 (Figure 2G,H). Similarly, phosphorylation of p38 MAPK, JNK, and ERK1/2 was elevated in the HFD group, whereas the WPR-H group exhibited lower phosphorylation levels of these MAPKs (Figure 2I,J).

### 3.3. Effects of WPR on the Mitochondrial Biogenesis and Protein Synthesis and Degradation in the GAS of Mice with OIMA

**Figure 3:**
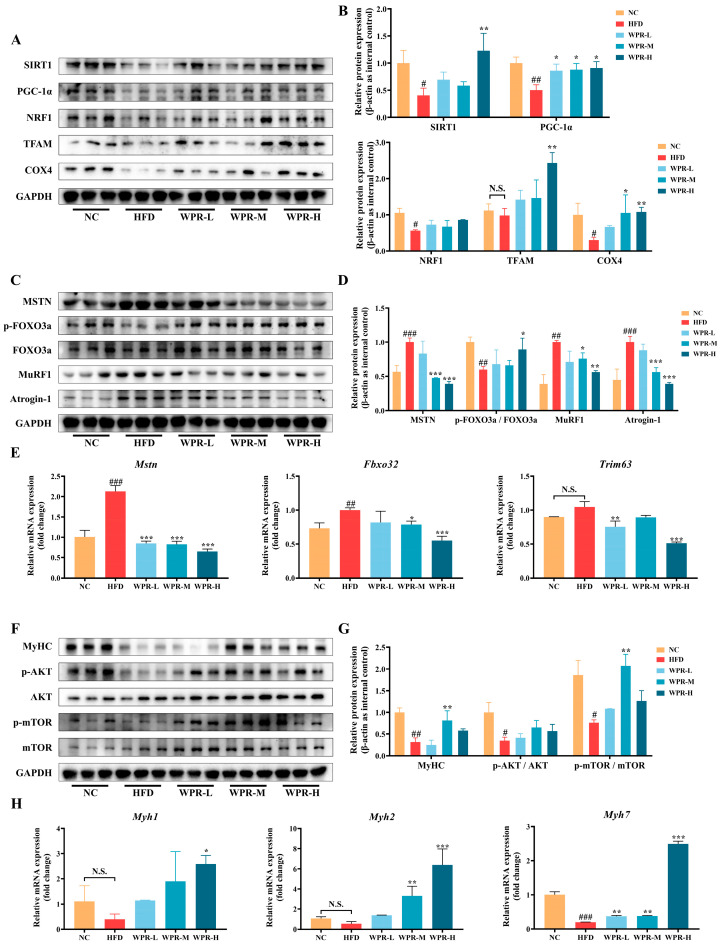
WPR administration improves the mitochondrial biogenesis and anabolic-catabolic balance in the GAS of mice with OIMA. (**A**,**B**) Representative Western blot images (**A**) and quantitative analyses (**B**) of SIRT1, PGC-1α, NRF1, TFAM, and COX4. (**C**,**D**) Representative Western blot images (**C**) and quantitative analyses (**D**) of MSTN, *p*-/total FOXO3a, MuRF1, and Atrogin-1. (**E**) Relative mRNA expression levels of *Mstn*, *Fbxo32*, and *Trim63* in the GAS. (**F**,**G**) Representative Western blot images (**F**) and quantitative analyses (**G**) of MyHC, p-/total AKT or mTOR. (**H**) Relative mRNA expression levels of *Myh1*, *Myh2*, and *Myh7* in the GAS. All data are presented as the mean ± SD (*n* = 3). The *p* values were defined as follows: ^#^ *p* < 0.05, ^##^ *p* < 0.01, and ^###^ *p* < 0.001 vs. the NC group; * *p* < 0.05, ** *p* < 0.01, and *** *p* < 0.001 vs. the HFD group; N.S., no significant (*p* ≥ 0.05). NC: normal control; HFD: high-fat diet; WPR: water extract of Polygonati Rhizoma.

To examine the effect of WPR on the changes in key regulators related to mitochondrial biogenesis, protein degradation, and anabolic signaling in muscle tissues under obese conditions, the expression of representative proteins and mRNAs was analyzed. As shown in Figure 3A,B, the HFD group exhibited reduced expression of mitochondrial biogenesis-related proteins, including SIRT1, PGC-1α, NRF1, TFAM, and COX4. In contrast, WPR-treated groups showed higher expression levels of these proteins, with significant changes observed in the WPR-H group.

Next, changes in protein degradation-related signaling were evaluated. As shown in Figure 3C,D, the HFD group displayed elevated protein levels of MSTN, MuRF1, and Atrogin-1, together with reduced FOXO3a phosphorylation. Compared with the HFD group, WPR-treated groups exhibited lower levels of MSTN, MuRF1, and Atrogin-1 and higher FOXO3a phosphorylation, particularly in the WPR-H group. Consistently, qRT-PCR analysis demonstrated that the mRNA expression levels of *Mstn*, *Fbxo32*, and *Trim63* were upregulated in the HFD group but significantly reduced in the WPR-treated groups (Figure 3E).

We further analyzed the changes in anabolic signaling and muscle structural proteins. As shown in Figure 3F,G, the HFD group exhibited reduced MyHC expression and lower phosphorylation levels of AKT and mTOR. In contrast, WPR-treated groups showed higher MyHC expression and increased phosphorylation of AKT and mTOR, with a significant elevation of MyHC and p-mTOR levels observed in the WPR-M group. Consistent with the protein changes, qRT-PCR analysis showed downregulation of the mRNA levels of muscle fiber-type markers *Myh1*, *Myh2*, and *Myh7* in the HFD group, with *Myh7* exhibiting a statistically significant reduction. However, WPR-treated groups exhibited increased expression of these markers, particularly in the WPR-H group (Figure 3H).

### 3.4. Effects of WPR on PA-Induced Myotube Atrophy in C2C12 Cells

To establish a reliable in vitro model of muscle atrophy, differentiated C2C12 myotubes (day 5) were treated with graded concentrations of PA for 24 h. As a result, PA at 0.5 mM markedly reduced the cell viability to 73.2% compared with the NC group (Figure S3A) and was accompanied by reduced protein expression of MyHC and Myogenin and increased levels of MSTN, Atrogin-1, and MuRF1 (Figure S3B,C). From this result, PA at 0.5 mM for 24 h was used to prepare a stable lipotoxic myotube atrophy model.

**Figure 4:**
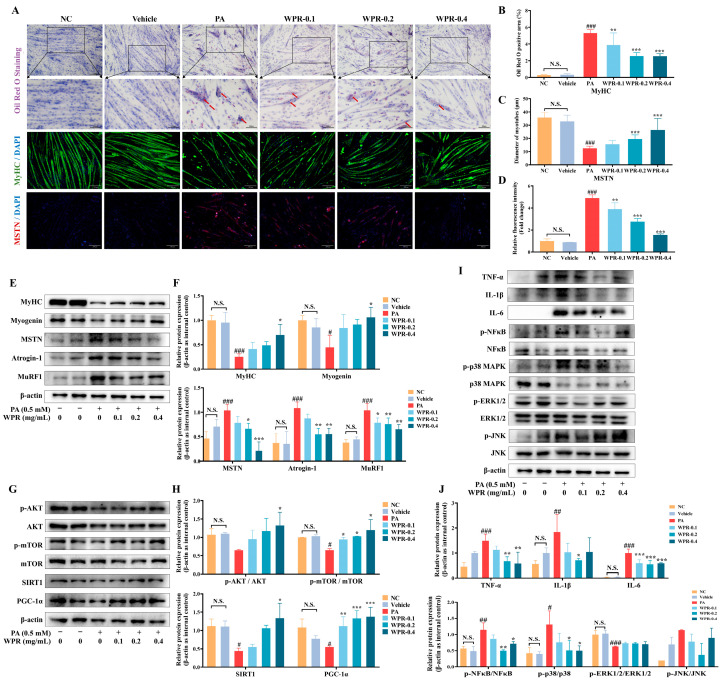
WPR treatment attenuates PA-induced myotube atrophy in C2C12 cells. (**A**–**D**) Representative images (**A**) and the quantitative analyses (**B**) of Oil Red O staining. Red arrows indicate regions of intense Oil Red O staining, representing localized lipid accumulation within cells. Representative ICC images (**A**) and quantitative analyses of MyHC (**C**) and MSTN (**D**) in PA-stimulated C2C12 myotubes with or without WPR treatment. (**E**–**J**) Representative Western blot images (**E**,**G**,**I**) and quantitative analyses (**F**,**H**,**J**) of MyHC, Myogenin, MSTN, Atrogin-1, MuRF1, the phosphorylation of AKT and mTOR, SIRT1, PGC-1α, TNF-α, IL-1β, IL-6, and the phosphorylation of NF-κB, ERK1/2, JNK, and p38 MAPK in PA-stimulated C2C12 myotubes with or without WPR treatment. All data are presented as the mean ± SD (*n* = 3). The *p* values were defined as follows: ^#^ *p* < 0.05, ^##^ *p* < 0.01 and ^###^ *p* < 0.001, vs. the NC group. * *p* < 0.05, ** *p* < 0.01 and *** *p* < 0.001 vs. the PA group. N.S., no significant (*p* ≥ 0.05). NC: normal control; PA: palmitic acid; WPR: water extract of Polygonati Rhizoma.

Next, changes in PA-induced myotube atrophy following WPR treatment were evaluated. WPR significantly improved the viability of PA-treated C2C12 myotubes (Figure S5A) and dose-dependently reduced the intracellular lipid accumulation (Figure 4A,B). Immunocytochemistry further showed that PA exposure led to suppression of MyHC expression (Figure 4A,C) and upregulation of MSTN (Figure 4A,D), both of which were dose-dependently reversed by WPR treatment. Consistently, Western blot analysis (Figure 4E,F) showed that PA stimulation strongly reduced the protein expression of MyHC and Myogenin while elevating the protein levels of MSTN and Atrogin-1. In contrast, WPR pretreatment (0.4 mg/mL) increased MyHC and Myogenin levels and reduced the PA-induced elevations of Atrogin-1, MuRF1, and MSTN.

We further examined changes in anabolic signaling and mitochondrial biogenesis-related markers in PA-stimulated C2C12 myotubes. The results showed that the phosphorylation levels of AKT and mTOR, as well as the protein expression of SIRT1 and PGC-1α, were markedly reduced in the PA group, indicating impaired anabolic signaling and mitochondrial biogenesis (Figure 4G,H). However, WPR treatment at 0.4 mg/mL significantly increased the AKT and mTOR phosphorylation and increased the protein levels of SIRT1 and PGC-1α.

In addition, inflammatory and stress-related signaling molecules were assessed. PA stimulation increased the protein levels of TNF-α, IL-1β, and IL-6, along with increased phosphorylation of NF-κB p65 and p38 MAPK in C2C12 myotubes compared with the NC group (Figure 4I,J). However, WPR pretreatment significantly reduced these PA-induced increases in inflammatory cytokine production and stress kinase activation.

### 3.5. Effect of WPR on LPS-Induced Myotube Atrophy in C2C12 Cells

**Figure 5:**
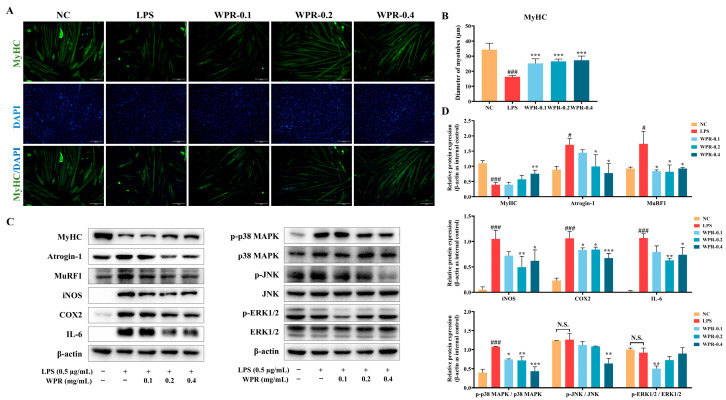
WPR treatment attenuates LPS-induced myotube atrophy in C2C12 cells. (**A**,**B**) Representative ICC images (**A**) and quantitative analysis (**B**) of MyHC in LPS-stimulated C2C12 myotubes. (**C**,**D**) Representative Western blot images (**C**) and quantitative analyses (**D**) of MyHC, Atrogin-1, MuRF1, iNOS, COX-2, IL-6, and the phosphorylation of p38 MAPK, JNK, and ERK1/2 in LPS-stimulated C2C12 myotubes with or without WPR treatment. All data are presented as the mean ± SD (*n* = 3). The *p* values were defined as follows: ^#^ *p* < 0.05 and ^###^ *p* < 0.001 vs. the NC group; * *p* < 0.05, ** *p* < 0.01, and *** *p* < 0.001 vs. the LPS group; N.S., no significant (*p* ≥ 0.05). NC: normal control; LPS: lipopolysaccharide; WPR: water extract of Polygonati Rhizoma.

To establish an inflammation-mediated myotube atrophy model, differentiated C2C12 myotubes were treated with LPS. As shown in Figure S4A–C, treatment with LPS at 1 μg/mL markedly suppressed the MyHC protein expression and increased the protein levels of MSTN, Atrogin-1, and COX-2 in C2C12 myotubes without significantly decreasing cell viability. Based on these results, LPS (1 μg/mL, 24 h) was selected to induce an inflammatory myotube atrophy model. Then, differentiated C2C12 myotubes (day 5) were treated with LPS with or without WPR pretreatment. As a result, WPR treatment at 0.1, 0.2, and 0.4 mg/mL did not decrease cell viability in LPS-stimulated C2C12 myotubes (Figure S5B). ICC analysis showed that LPS stimulation reduced the expression of MyHC and the myotube diameter (Figure 5A). While under WPR treatment, myotube diameter was markedly increased, and the expression of MyHC was partially restored (Figure 5B).

Western blot analysis (Figure 5C,D) further showed that LPS stimulation decreased MyHC expression and increased Atrogin-1 and MuRF1 levels, whereas pretreatment with WPR (0.4 mg/mL) resulted in higher MyHC levels and lower Atrogin-1 and MuRF1 levels compared with the LPS group. Meanwhile, LPS increased the expression of iNOS, COX-2, and IL-6 and enhanced p38 MAPK phosphorylation, while WPR pretreatment (0.4 mg/mL) reduced these inflammatory expressions and phosphorylation signals.

### 3.6. Effects of WPR on the Muscle Differentiation in C2C12 Cells

**Figure 6:**
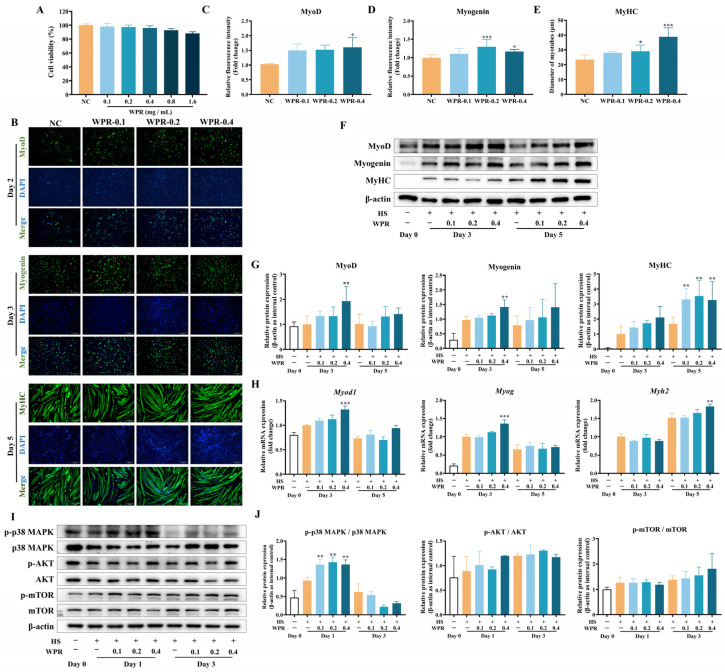
WPR treatment promotes muscle differentiation in C2C12 cells. (**A**) Cell viability of C2C12 cells under WPR treatment. (**B**–**E**) Representative ICC images (**B**) and quantitative analyses of MyoD (**C**), Myogenin (**D**), and MyHC (**E**) in C2C12 cells with or without WPR treatment. (**F**,**G**) Representative Western blot images (**F**) and quantitative analyses (**G**) of MyoD, Myogenin, and MyHC on days 0, 3, and 5 in C2C12 cells with or without WPR treatment. (**H**) The mRNA expression levels of *Myod1*, *Myog*, and *Myh2* in C2C12 cells with or without WPR treatment on days 0, 3, and 5 during differentiation. (**I**,**J**) Representative Western blot images (**I**) and quantitative analyses (**J**) of the phosphorylation and total protein levels of p38 MAPK, AKT and mTOR on day 0, 1, and 3 in C2C12 cells with or without WPR treatment. All data are presented as the mean ± SD (*n* = 3). The *p* values were defined as follows: * *p* < 0.05, ** *p* < 0.01, and *** *p* < 0.001 vs. the NC group. NC: normal control; WPR: water extract of Polygonati Rhizoma.

To explore whether WPR regulates myogenic differentiation, C2C12 myoblasts were induced to differentiate with medium containing HS in the presence of WPR. As shown in Figure 6A, WPR treatment at 0.4 mg/mL did not show a significant decrease in cell viability (96.2% of the NC group) in C2C12 cells.

During the differentiation process, ICC analysis (Figure 6B) showed that WPR treatment increased the proportion of MyoD^+^ cells on day 2 (Figure 6C) and Myogenin^+^ cells on day 3 (Figure 6D). By day 5, WPR-treated cells exhibited thicker and more fused myotubes, together with an increased proportion of MyHC-positive cells (Figure 6E). Consistently, Western blot analysis (Figure 6F,G) showed higher protein expressions of MyoD and Myogenin on day 3 and increased MyHC expressions on day 5 in the WPR-treated groups. In line with these protein changes, qRT-PCR analysis demonstrated increased mRNA expression of *Myod1* and *Myog* in the WPR-treated groups at the early differentiation stage (day 3) and increased Myh2 expression at the late stage (day 5) (Figure 6H).

To further examine changes in differentiation-related signaling pathways, phosphorylation of p38 MAPK was assessed on day 1, and phosphorylation of AKT and mTOR was assessed on day 3 (Figure 6I,J). Notably, p38 MAPK phosphorylation on day 1 was higher in the WPR-treated groups during differentiation, whereas the phosphorylation levels of AKT and mTOR showed no significant differences between groups.

## 4. Discussion

Sarcopenic obesity (SO) is a metabolic myopathy characterized by reduced skeletal muscle mass and strength in the context of excessive adiposity, and its prevalence and progression track closely with rising obesity rates and population aging [24]. Polygonati Rhizoma (PR) is commonly used as a representative Yin-nourishing herb in traditional Chinese and Korean medicine and also widely used as part of the diet (e.g., porridge, tea, medicinal wines) [13]. It was reported that the aqueous extract of PR ameliorates HFD-induced obesity and hepatic injury via modulation of the gut microbiota and lipid-metabolic gene [16], and the polysaccharides of PR have benefits on muscle atrophy associated with aging or cachexia [18,19,20]. Based on these pharmacological properties, WPR may have the potential to exert beneficial effects on obesity-related skeletal muscle atrophy. Accordingly, in this study, we explored the effects and potential mechanisms of WPR on obesity-related muscle atrophy through complementary in vivo and in vitro experiments.

Hypercaloric diets, dysregulated glucose-lipid metabolism, insulin resistance, and abnormal expansion or redistribution of adipose depots are key drivers of obesity and muscle atrophy [2]. In people with SO, a visceral obesity phenotype is associated with higher inflammatory and fracture risk and poorer metabolic status compared with a subcutaneous obesity phenotype [25]. Visceral adipose tissue exhibits higher metabolic and pro-inflammatory activity than subcutaneous fat, releasing TNF-α, IL-6, MCP-1, and large amounts of free fatty acids that enter the portal circulation and subsequently aggravate dyslipidemia, systemic inflammation, and insulin resistance in skeletal muscle, and subsequently drive the formation of a lipotoxic and pro-inflammatory microenvironment in skeletal muscle. This pathological milieu collectively suppresses mitochondrial biogenesis and protein synthesis while accelerating protein degradation, ultimately leading to muscle fiber atrophy, disrupted muscle fiber organization, and declines in muscle mass, strength, and endurance [26,27].

In our study, we established an OIMA mouse model, which is characterized by obesity and declines in muscle mass, strength, and endurance. However, WPR administration significantly improved the muscle strength and endurance in OIMA mice and increased the muscle mass of GAS and SOL. Furthermore, we observed that WPR administration resulted in a more orderly arrangement of muscle fibers, increased mean CSA, and a shift in CSA distribution from smaller to larger fibers in the GAS. These findings suggest that WPR attenuated obesity-related declines in skeletal muscle mass and function and was associated with improved structural integrity of skeletal muscle in OIMA mice.

In addition, the administration of WPR for 6 weeks in OIMA mice reduced the excessive body weight gain and fasting hyperglycemia, improved glucose tolerance and insulin resistance, reduced fat accumulation in iWAT and eWAT, and restored BAT (Figure S1). WPR administration also improved the abnormal serum biochemical parameters and mitigated liver and kidney injury (Figure S2). These results suggest WPR administration was also associated with improvements in serum biochemical parameters and markers of liver and kidney injury. Although these systemic metabolic changes were not primary endpoints of the present study, they may contribute to a more favorable skeletal muscle microenvironment under obesity.

Skeletal muscle integrity is preserved through a tightly regulated balance between anabolic and catabolic processes. Under obesity and insulin resistance, IRS-1/2–PI3K–AKT signaling is restrained by lipotoxic species (diacylglycerol and ceramides) and inflammatory cytokines, suppressing the rapamycin complex 1 (mTORC1)-p70S6K/4E-BP1 translational axis [28]. Concurrently, the decrease of AKT phosphorylation relieves the inhibition of the FOXO transcription factors, up-regulates Atrogin-1/MAFbx and MuRF1, and thereby enhances the UPS-mediated degradation of proteins, ultimately disturbing the balance between protein synthesis and degradation. Moreover, it has been reported that mitochondrial biogenesis is markedly down-regulated in the HFD-induced obesity mouse model and the PA-induced myotube atrophy model in cells, which is in close association with the AKT dysregulation and altered AMPK/SIRT1 activity [10,29]. Our data suggested that WPR treatment was associated with increased MyHC expression and activation of the AKT/mTOR signaling axis in the GAS of OIMA mice, accompanied by reduced expression of MSTN, Atrogin-1, and MuRF1 and increased levels of mitochondrial biogenesis-related proteins, including PGC-1α, NRF1, TFAM, and COX4. Similar results were observed in C2C12 cells, in which WPR was associated with enhanced myogenic marker expression, increased AKT/mTOR phosphorylation, and suppression of atrogene expression under PA- or LPS-induced atrophic conditions. Collectively, these exploratory observations are consistent with the possibility that modulation of UPS-driven proteolysis, anabolic AKT/mTOR signaling, and mitochondrial biogenesis may contribute to the anti-atrophic effects of WPR, although causal relationships were not directly validated in the present study.

In obesity and insulin resistance, overnutrition and ectopic lipid deposition increase mitochondrial ROS (mtROS), leading to oxidative damage to proteins, lipids, and mitochondrial DNA, accompanied by depletion of the antioxidant defense system [30]. In addition, visceral adipose tissue and intermuscular adipose tissue release TNF-α, IL-6, and IL-1β, shaping a sustained inflammatory microenvironment through systemic circulation and paracrine signaling in skeletal muscle [10]. During this process, excessive mtROS further intensifies inflammation by activating NF-κB and JNK signaling, which in turn drives the persistent elevation of pro-inflammatory cytokines [11]. This, in turn, induced mitochondrial damage and promoted the expression of atrophy-related genes, forming a vicious cycle of oxidative stress, inflammation, mitochondrial dysfunction, and lipotoxicity [28]. Meanwhile, MAPKs, particularly p38 MAPK, act as upstream signaling hubs in various muscle atrophy models. p38 MAPK is involved in TNF-α–induced upregulation of MuRF1 and Atrogin-1 and is also associated with ROS accumulation and cytokine exposure [31,32,33]. Studies have shown that pharmacological inhibition or genetic deletion of p38α MAPK markedly attenuates denervation-induced muscle atrophy [34]. As part of the exploratory mechanistic analyses in the present study, WPR treatment was associated with reduced MDA levels, increased SOD activity, and decreased mRNA and protein expression of TNF-α, IL-1β, and IL-6 in the GAS of OIMA mice, accompanied by suppression of the TLR4/NF-κB and MAPK signaling pathways. Consistent with these in vivo observations, WPR treatment in C2C12 cells was associated with attenuation of PA-induced production of TNF-α, IL-1β, and IL-6 and reduced phosphorylation of NF-κB and p38 MAPK. In addition, in LPS-induced myotube atrophy models, WPR treatment was associated with reduced expression of iNOS, COX-2, and IL-6, along with suppression of p38 MAPK phosphorylation. Collectively, these exploratory observations are consistent with a potential association between WPR treatment and modulation of oxidative stress- and inflammation-related signaling pathways in obesity-related muscle atrophy.

**Figure 7:**
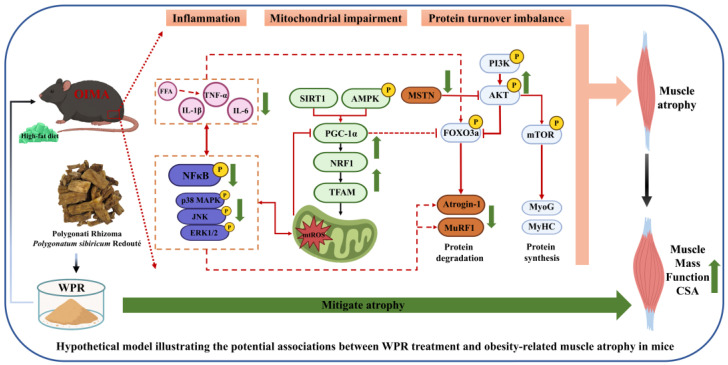
Hypothetical model illustrating the potential associations between WPR treatment and obesity-related muscle atrophy in mice. Green arrows indicate changes following WPR treatment. OIMA: obesity-induced muscle atrophy mouse model; WPR: water extract of Polygonati Rhizoma; FFA: free fatty acid; mtROS: mitochondrial reactive oxygen species; CSA: cross-sectional area.

Notably, p38 MAPK has also been identified as a positive regulator of muscle satellite cell differentiation. Mild and transient activation of p38 MAPK during the early phase of myogenesis can promote the activation of MyoD/MyoG and MEF2, as well as the AKT–mTOR pathway, thereby enhancing protein translation and myofiber maturation [33,34,35,36,37]. However, under stress or inflammatory conditions, sustained activation of p38 MAPK has been associated with impaired muscle differentiation and muscle atrophy. In this study, the regulatory effects of WPR on p38 MAPK seem to be context-dependent. Under lipotoxic or inflammatory challenge, such as PA- or LPS-induced myotube atrophy and OIMA muscle tissues, WPR treatment was associated with attenuation of excessive p38 MAPK phosphorylation, accompanied by suppression of inflammatory signaling and improvements in muscle atrophy–related phenotypes. However, in the HS-induced myogenic differentiation of C2C12 myoblasts, WPR treatment was associated with increased expression of MyoD and Myogenin and a transient enhancement of p38 MAPK phosphorylation during the early differentiation phase (day 1). These results suggest that WPR may not function as a unidirectional regulator of p38 MAPK signaling, rather, it may restore the physiological balance of p38 MAPK signaling in a context-dependent manner. Nevertheless, it should be noted that our current data only show the relation between WPR and p38 MAPK modulation during myogenesis, but the direct causality remains to be established through further investigation. Furthermore, the p38 MAPK activation may not represent the primary driving mechanism of the promotive effects of WPR on myogenic differentiation, other signaling pathways, such as the AKT/mTOR axis or mitochondrial metabolic remodeling, may also be involved. Our findings are all summarized in a hypothetical model (Figure 7), which is intended to integrate the potential associations between WPR treatment and obesity-related muscle atrophy in mice.

Consistent with the exploratory and hypothesis-generating nature of the present study, several limitations should be acknowledged when interpreting the anti-obesity-related muscle atrophy effects of WPR observed in the in vivo and in vitro models. First, the group size in our study was not determined based on predefined effect sizes or formal power calculations. Accordingly, this study was not intended as a statistically powered hypothesis-testing investigation, and the observed *p*-values should not be interpreted as providing definitive quantitative estimates of effect magnitude or biological certainty. In addition, although randomization, allocation concealment, and blinded outcome assessment were implemented to minimize bias, complete elimination of bias cannot be guaranteed due to practical constraints inherent to animal experiments. Second, WPR is an aqueous extract (yield: 67.32%) including multi-components. In the LC–MS analysis of WPR (Figure S6, Table S3), amino acids, small peptides, saponins, flavonoids/phenolic compounds, and oligosaccharide/polysaccharide fragments were identified. However, detailed quantification and attribution of the polysaccharides and individual components were not performed. Thus, the relative contributions of individual components to anti-obesity-related muscle atrophy activity remain unclear. Third, WPR administration increased the transcription of Myh1, Myh2, and Myh7 in the GAS of OIMA mice. However, the potential effects of WPR on the composition and transition between oxidative and glycolytic muscle fiber types remain to be further investigated. Finally, our study suggests that WPR may promote myogenic differentiation from myoblasts into myotubes through the regulation of the p38 MAPK/MyoD/Myogenin axis at the early phase. However, it should be supported by evidence from the use of p38 MAPK inhibitors or genetic loss-of-function approaches. Therefore, the current evidence supports only a correlative relationship. Future studies should incorporate pharmacological or genetic inhibition to verify the necessity and specificity.

## 5. Conclusions

In conclusion, our study suggests that WPR may attenuate obesity-related muscle atrophy and functional impairment in OIMA model, and that these effects are associated with changes in oxidative stress, inflammatory responses, mitochondrial biogenesis, and protein metabolic balance in skeletal muscle. However, it is important to note that our findings are based on preclinical models, and WPR is a multi-component extract with a complicated chemical composition. Therefore, the underlying causal mechanisms of the regulatory effects of WPR on skeletal muscle mass and function remain to be further validated to clarify the biological relevance and potential application value of WPR in obesity-related muscle atrophy.

## Data Availability

All datasets supporting the conclusions of this research are contained within the article and its Supplementary Files, and additional information can be provided by the authors upon reasonable request.

## Supplementary Materials

The following supporting information can be downloaded at: https://www.mdpi.com/article/10.3390/nu18030429/s1, Figure S1: WPR administration ameliorates the glucose-lipid metabolic dysfunction in mice with OIMA; Figure S2: WPR administration alleviates HFD-induced hepatic and renal injury in OIMA mice; Figure S3: Establishment of PA-induced myotube atrophy model in C2C12 myotubes; Figure S4: Establishment of LPS-induced myotube atrophy model in C2C12 myotubes; Figure S5: WPR has no significant cytotoxic effect on C2C12 myotubes under PA or LPS stimulation; Figure S6: Results of UHPLC-Q-TOF-MS/MS analysis. Table S1: Information of primers used in the qRT-PCR analysis; Table S2: Information of antibodies used in the western blot analysis; Table S3: Chemical information of WPR identified by the UHPLC-Q-TOF-MS/MS analysis.
