# Supplementary material for: Water Extract of Polygonati Rhizoma Ameliorates Obesity-Related Skeletal Muscle Atrophy in Mice and C2C12 Myotubes
Source: Nutrients. 2026 Jan 28;18(3):429. doi: 10.3390/nu18030429 (PMC12899732; doi:10.3390/nu18030429)
Supplement: Supplementary file 1 [file nutrients-18-00429-s001.zip › nutrients-4076026-supplementary.pdf]

Table S1. Information of primers used in the qRT-PCR analysis

Gene	Accession No.	Primer (5′-3′)	Sequences
Myod1	NM_010866.2	Forward	CTCTGATGGCATGATGGATT
		Reverse	CGAAAGGACAGTTGGGAAGA
MyoG	NM_031189.2	Forward	CTTGCTCAGCTCCCTCAACC
		Reverse	GGTGTTAGCCTTATGTGAATGG
Myh2	NM_001039545.2	Forward	AGTTCCGCAAGATCCAGCAC
		Reverse	CGGGACAGCCTTACTCTTCG
Myh1	NM_030679.2	Forward	GAAGTTGCATCCCTAAAGGCAG
		Reverse	GCTTGTTCTGAGCCTCGATTC
Fbxo32	NM_026346.3	Forward	TTCAGCAGCCTGAACTACGA
		Reverse	TGAAAGCTTCCCCCAAAGTA
Trim63	NM_001039048.2	Forward	TGAGGTGCCTACTTGCTCCT
		Reverse	TCACCTGGTGGCTATTCTCC
Mstn	NM_010834.3	Forward	ACGCTACCACGGAAACAATC
		Reverse	GGAGTCTTGACGGGTCTGAG
Tnf	NM_013693.3	Forward	GTCCCCAAAGGGATGAGAAGT
		Reverse	TTTGCTACGACGTGGGCTAC
IL-1β	NM_008361.4	Forward	TGCCACCTTTTGACAGTGATG
		Reverse	AAGGTCCACGGGAAAGACAC

Table S2. Information of antibodies used in the western blot analysis

Type	Target Protein	Catalog Number	Company	Dilution Ratio
Primary	β-actin	#A5316	Abcam	1:1000
Primary	GAPDH	#PA0018	Thermo Fisher	1:1000
Primary	MyoD	#sc-377460	Santa Cruz	1:1000
Primary	MyHC	#sc-376157	Santa Cruz	1:1000
Primary	Myogenin	#sc-52903	Santa Cruz	1:1000
Primary	MuRF1	#BS-2539R	Bioss	1:1000
Primary	Atrogin-1	#PA5-91959	Thermo Fisher	1:1000
Primary	FxoO3a	#PA5-27145	Thermo Fisher	1:1000
Primary	Phospho-FoxO3a	#PA5-36816	Thermo Fisher	1:1000
Primary	Myostatin	BS-1288r	Bioss	1:1000
Primary	SIRT1	BS-0921r	Bioss	1:1000
Primary	PGC1α	#2178s	CST	1:1000
Primary	NRF1	#69432s	CST	1:1000
Primary	TFAM	#PA5-68789	Thermo Fisher	1:1000
Primary	COX4	#BS-10257R	Bioss	1:1000
Primary	AKT	#9272s	CST	1:1000
Primary	p-AKT	#9271s	CST	1:1000
Primary	mTOR	#2972s	CST	1:1000
Primary	p-mTOR	#5536s	CST	1:1000
Primary	iNOS	#13120s	CST	1:1000
Primary	COX-2	#12282s	CST	1:1000
Primary	TLR4	#48-2300	Thermo Fisher	1:1000
Primary	NFκB p65	#4764s	CST	1:1000
Primary	Phospho-NFκB p65	#3033s	CST	1:1000
Primary	IκBα	#4812s	CST	1:1000
Primary	Phospho-IκBα	#2859s	CST	1:1000
Primary	JNK	#9252s	CST	1:1000
Primary	Phospho-JNK	#9251s	CST	1:1000
Primary	ERK1/2	#9102s	CST	1:1000
Primary	Phospho-ERK1/2	#9101s	CST	1:1000
Primary	p38 MAPK	#9212s	CST	1:1000
Primary	Phospho-p38 MAPK	#9211s	CST	1:1000
Primary	TNF-α	#11948s	CST	1:1000
Primary	IL-6	#12912S	CST	1:1000
Primary	IL-1β	#12426s	CST	1:1000
Secondary	Goat anti-mouse IgG-HRP	#1706516	Bio-Rad	1:5000
Secondary	Goat anti-rabbit IgG-HRP	#1706515	Bio-Rad	1:5000
Secondary	Alexa Fluor® 488 goat anti-mouse IgG	#A11001	Thermo Fisher Scientific	1:1000
Secondary	Alexa Fluor® 594 anti-rabbit IgG	#8889	Thermo Fisher Scientific	1:1000

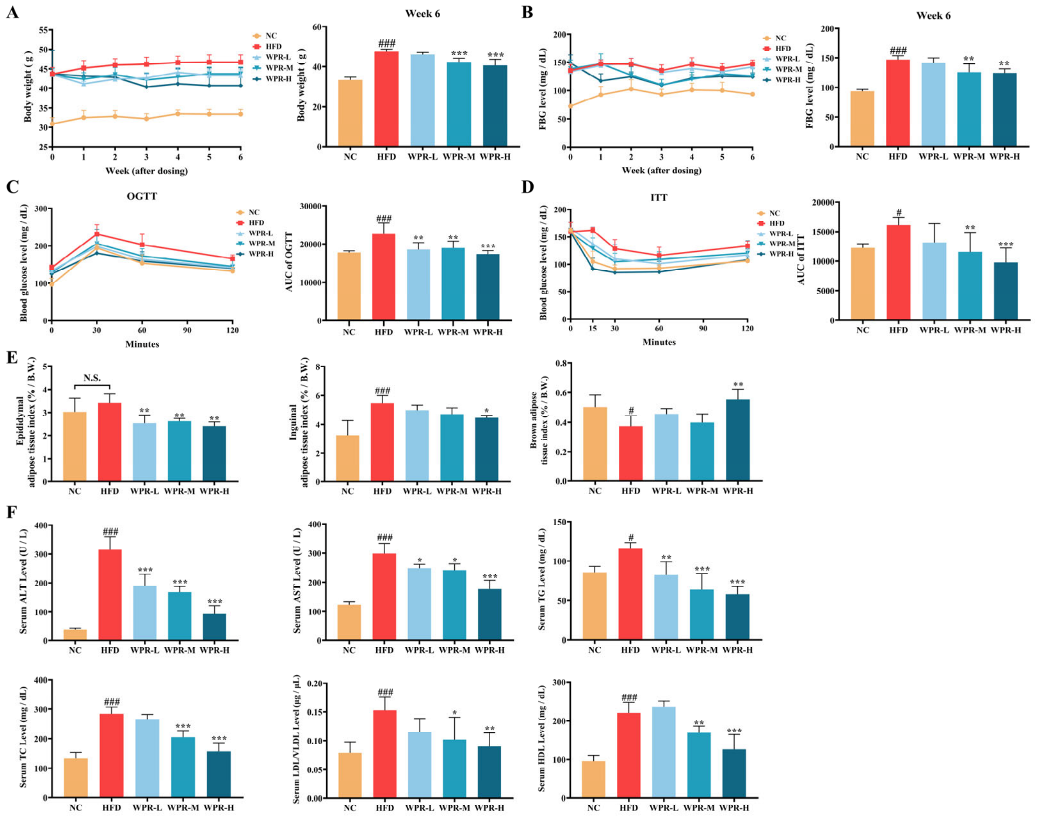

Figure S1. WPR administration ameliorates the glucose-lipid metabolic dysfunction in mice with OIMA. (A) Comparison of body weights among groups. (B) Comparison of the FBG levels among groups. (C–D) Blood glucose levels and the AUC in each group in the OGTT (C) and ITT (D). (E) Mass indices of iWAT, eWAT, and BAT in each group. (F) Comparisons of the serum levels of ALT, AST, TG, TC, LDL/VLDL, and HDL among groups. All data are presented as the mean ± SD ($n = 6$). The p values were defined as follows: # $p < 0.05$ and ### $p < 0.001$ vs. the NC group; * $p < 0.05$, ** $p < 0.01$, and *** $p < 0.001$ vs. the HFD group. NC: normal control; HFD: high-fat diet; WPR: water extract of Polygonati Rhizoma; FBG: fasting blood glucose, OGTT: oral glucose tolerance test; ITT: insulin tolerance test; ALT: alanine aminotransferase; AST: aspartate aminotransferase; TG: triglycerides; TC: total cholesterol; LDL: low-density lipoprotein; HDL: high-density lipoprotein; AUC: area under the curve.

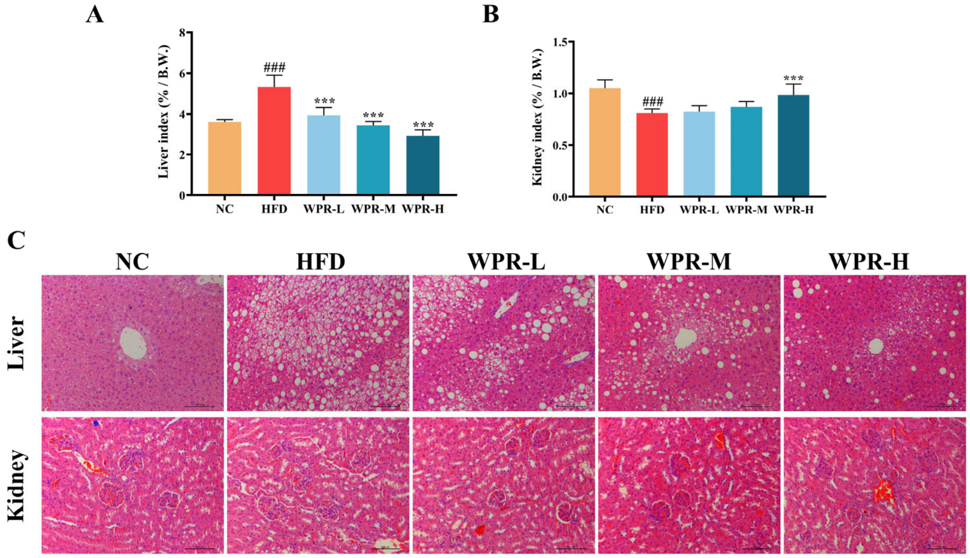

Figure S2. WPR administration alleviates HFD-induced hepatic and renal injury in OIMA mice. (A-B) Comparisons of liver (A) and kidney (B) index among all groups (normalized to body weight). (C) Representative images of H&E-stained sections of liver and kidney from each group. All data are presented as the mean ± SD (n = 6). The p values were defined as follows: ### $p < 0.001$ vs. the NC group; *** $p < 0.001$ vs. the HFD group. NC: normal control; HFD: high-fat diet; WPR: water extract of Polygonati Rhizoma.

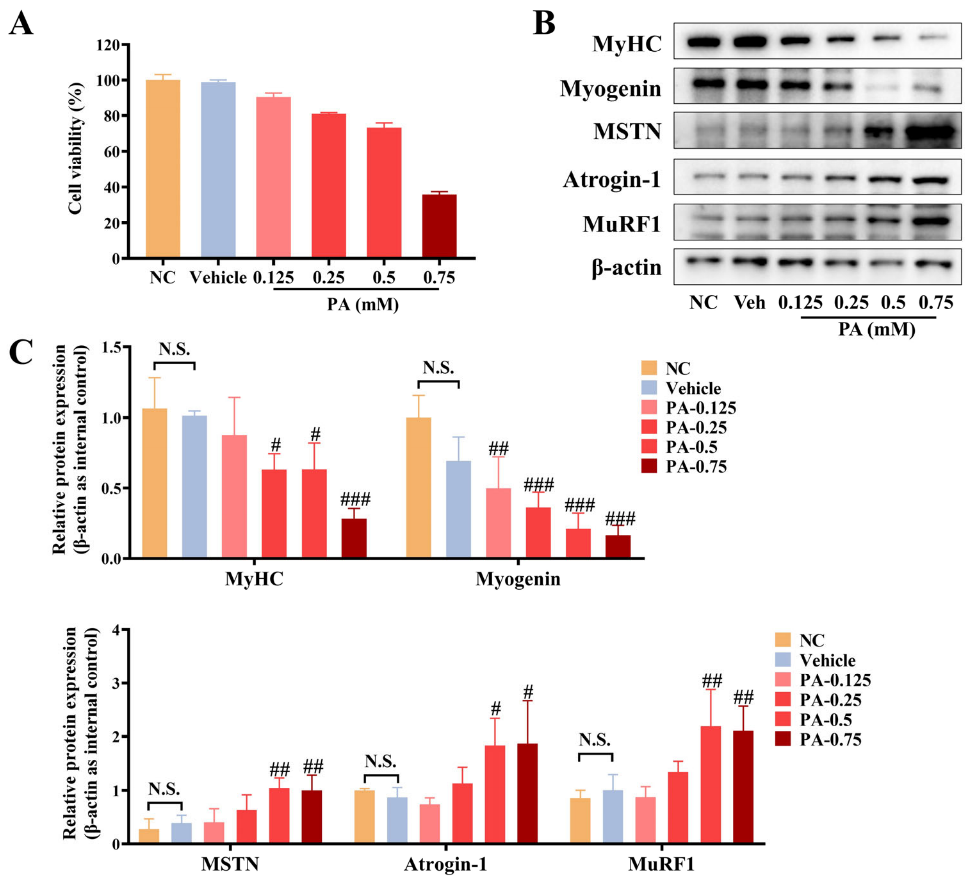

Figure S3. Establishment of PA-induced myotube atrophy model in C2C12 myotubes. (A) Cell viability of C2C12 cells after PA stimulation for 24 hr. (B-C) Representative western blot images (B) and quantitative analyses (C) of MyHC, Myogenin, MSTN, Atrogin-1, and MuRF1 in PA-stimulated C2C12 myotubes. All data are presented as the mean ± SD ($n = 3$). The p values were defined as follows: # $p < 0.05$, ## $p < 0.01$ and ### $p < 0.001$, vs. the NC group; N.S., no significant ($p \geq 0.05$). NC: normal control. PA: palmitic acid.

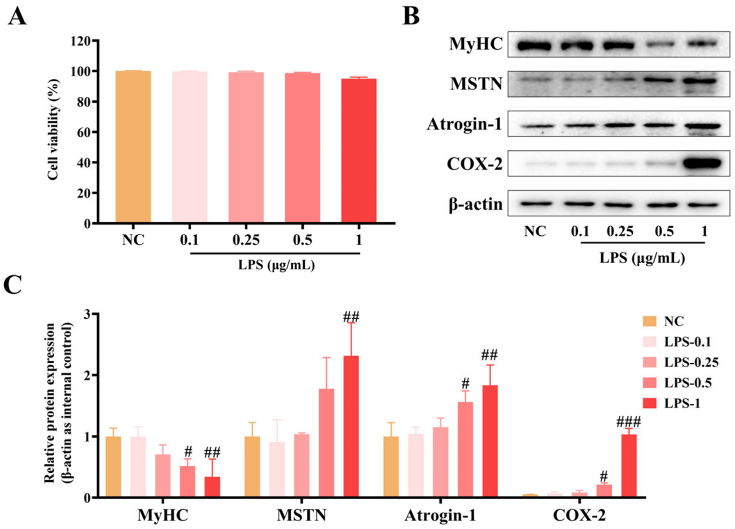

Figure S4. Establishment of LPS-induced myotube atrophy model in C2C12 myotubes. (A) Cell viability of C2C12 cells after LPS stimulation (μg/mL) for 24 hr. (B-C) Representative western blot images (B) and quantitative analyses (C) of MyHC, MSTN, Atrogin-1, and COX-2 in LPS-stimulated C2C12 myotubes. All data are presented as the mean ± SD ($n = 3$). The p values were defined as follows: # $p < 0.05$, ## $p < 0.01$ and ### $p < 0.001$, vs. the NC group. NC: normal control; LPS: lipopolysaccharide.

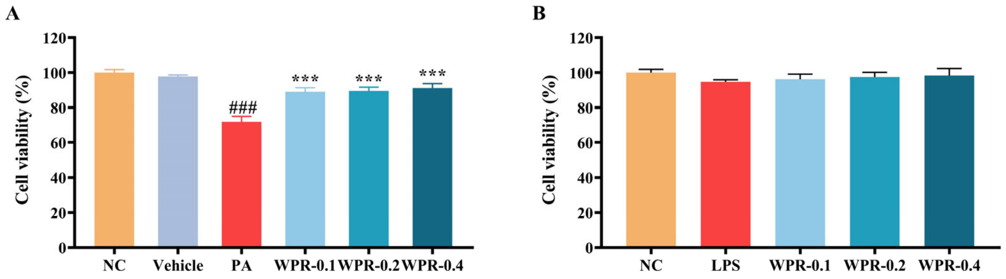

Figure S5. WPR has no significant cytotoxic effect on C2C12 myotubes under PA or LPS stimulation. (A) Effect of WPR on the cell viability of C2C12 cells under PA stimulation. (B) Effect of WPR on the cell viability of C2C12 cells under LPS stimulation. All data are presented as the mean ± SD (n = 3). The p values were defined as follows: ### $p < 0.001$ vs. the NC group; *** $p < 0.001$ vs. the PA group. NC: normal control; PA: palmitic acid; LPS: lipopolysaccharide; WPR: water extract of Polygonati Rhizoma.

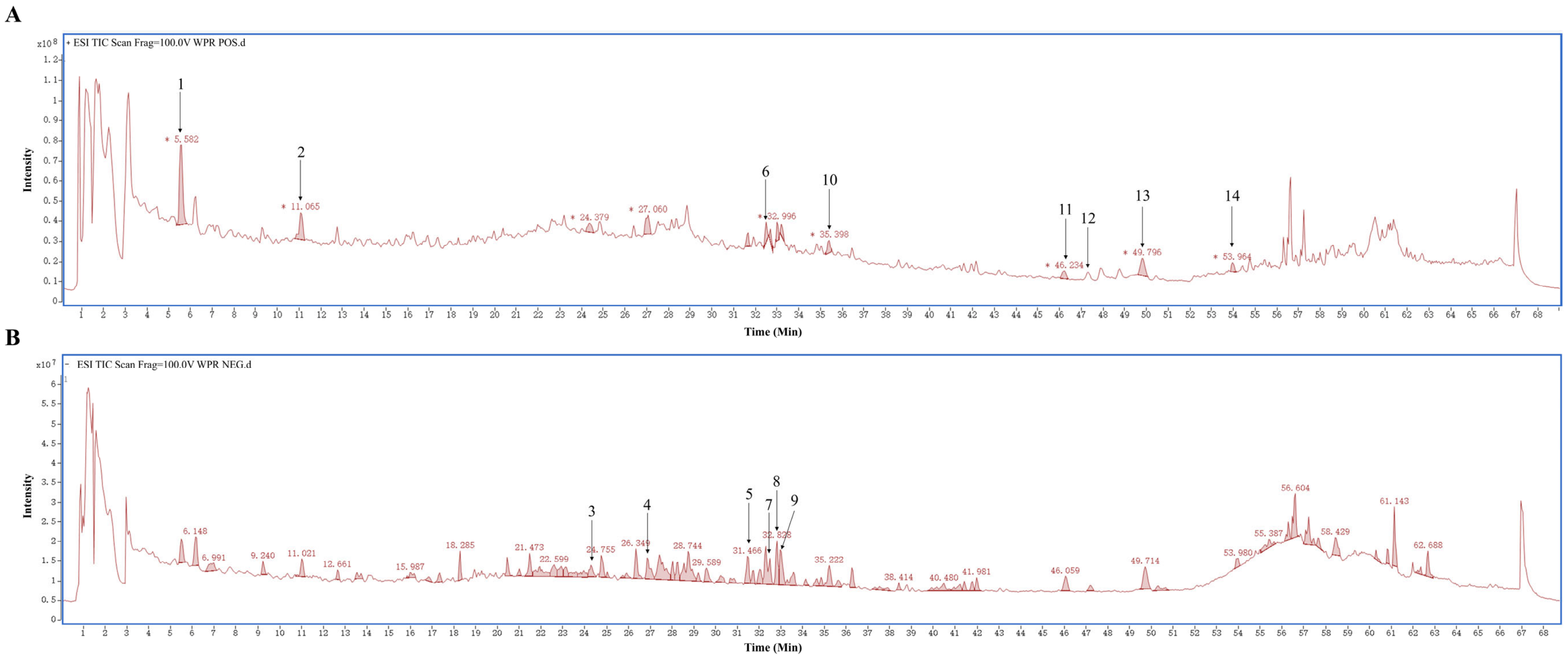

Figure S6. Results of UHPLC-Q-TOF-MS/MS analysis. (A) Total ion chromatograms of WPR in positive ion mode. (B) Total ion chromatograms of WPR in negative ion mode.

Table S3 Chemical information of WPR identified by the UHPLC-Q-TOF-MS/MS analysis

NO.	Chemical Compound	Formula	Retention time (min)	Measured (m/z)	Adduct Ions	ppm	MS²
1	L-Tryptophan	$C_{11}H_{12}N_2O_2$	5.582	205.0987	$[M+H]^+$	0.32	188.0713, 159.0920, 146.0605, 118.0653
				203.0832	$[M-H]^-$	0.94	203.0817, 186.0558, 159.0925, 142.0663, 116.0504
2	Val-Leu-Tyr	$C_{20}H_{31}N_3O_5$	11.065	394.2345	$[M+H]^+$	0.75	394.2331, 295.1649, 213.1596, 185.1646, 136.0751
				392.2190	$[M-H]^-$	0.28	392.2187, 228.1716, 180.0668, 115.0886
3	Pratioside B	$C_{51}H_{84}O_{25}$	24.286	1079.5287	$[M-H_2O+H]^+$	-4.32	1095.5227, 933.4726, 573.4948, 469.5797
				1095.5208	$[M-H]^-$	0.6	
4	Ophiopogonin A	$C_{50}H_{82}O_{22}$	26.865	1079.5300	$[M+HCOO]^-$	-0.16	1079.5286, 917.4708, 755.4207, 321.0616
5	Pratioside C	$C_{51}H_{82}O_{24}$	31.466	1137.5353	$[M+HCOO]^-$	0.66	1137.5337, 933.4710, 560.0017, 331.9605
6	Polygonatumoside F	$C_{51}H_{82}O_{23}$	32.499	1063.5330	$[M+H]^+$	-2.71	1063.5311, 739.4256, 577.37
7	Diuranthoside A	$C_{50}H_{82}O_{22}$	32.499	1079.5296	$[M+HCOO]^-$	1.46	1079.5280, 917.4733, 755.4229, 312.7298
8	Capsicoside E1	$C_{56}H_{92}O_{28}$	32.828	1195.5745	$[M-H_2O+H]^+$	-1.27	1211.5739, 1079.5569, 917.4526, 755.4215
				1211.5715	$[M-H]^-$	1.08	
9	Digalonin	$C_{56}H_{92}O_{28}$	32.969	1195.5709	$[M-H_2O+H]^+$	1.13	1211.5739,1079.5569,917.4526,755.4215,523.2905,250.1493
				1211.5713	$[M-H]^-$	-1.73	
10	Sibiricoside B	$C_{50}H_{80}O_{24}$	35.398	1047.5385	$[M-H_2O+H]^+$	-1.84	1047.5505, 885.4820, 577.3723, 415.3206, 253.1948
				1063.4968	$[M-H]^-$	-0.01	1063.5339, 902.4461, 607.3471, 221.0662
11	Odoratumone B	$C_{17}H_{16}O_5$	46.234	301.1076	$[M+H]^+$	0.32	301.1069, 195.0647, 107.0487
				299.0926	$[M-H]^-$	-0.89	299.0926, 193.0506, 149.0594, 121.0291, 107.0491

12	4′,5,7-Trihydroxy-6-methyl-8-methoxy-homoisoflavanone	$C_{18}H_{18}O_6$	47.313	331.1178	$[M+H]^+$	0.62	225.0761, 137.0601, 107.0494
				329.1029	$[M-H]^-$	-2.45	329.1033, 269.2132, 208.0374, 152.0475
13	P-hydroxyphenethyl trans-ferulate	$C_{18}H_{18}O_5$	49.796	315.1239	$[M+H]^+$	0.67	315.1238, 209.0817, 107.0497
				313.1085	$[M-H]^-$	-1.13	313.1078, 207.0662, 179.0711, 135.0454
14	2,3-Dihydro-5,7-dihydroxy-3-[(2-hydroxy 4-methoxyphenyl)methyl]-6,8-dimethyl-4H 1-benzopyran-4-one (ACI)	$C_{19}H_{20}O_6$	53.964	345.1328	$[M+H]^+$	-0.56	345.1337, 221.0814, 137.0602
				343.1182	$[M-H]^-$	-7.23	343.1184, 264.9932, 207.0672, 153.0558, 111.0454
